# Updates on the risk factors for latent tuberculosis reactivation and their managements

**DOI:** 10.1038/emi.2016.10

**Published:** 2016-02-03

**Authors:** Jing-Wen Ai, Qiao-Ling Ruan, Qi-Hui Liu, Wen-Hong Zhang

**Affiliations:** Department of Infectious Diseases, Huashan Hospital, Fudan University, Shanghai 200040, China

**Keywords:** Latent tuberculosis infection, risk factor, preventive treatment, regimen, reactivation

## Abstract

The preventive treatment of latent tuberculosis infection (LTBI) is of great importance for the elimination and control of tuberculosis (TB) worldwide, but existing screening methods for LTBI are still limited in predicting the onset of TB. Previous studies have found that some high-risk factors (including human immunodeficiency virus (HIV), organ transplantation, silicosis, tumor necrosis factor-alpha blockers, close contacts and kidney dialysis) contribute to a significantly increased TB reactivation rate. This article reviews each risk factor's association with TB and approaches to address those factors. Five regimens are currently recommended by the World Health Organization, and no regimen has shown superiority over others. In recent years, studies have gradually narrowed down to the preventive treatment of LTBI for high-risk target groups, such as silicosis patients, organ-transplantation recipients and HIV-infected patients. This review discusses regimens for each target group and compares the efficacy of different regimens. For HIV patients and transplant recipients, isoniazid monotherapy is effective in treating LTBI, but for others, little evidence is available at present.

## INTRODUCTION

The preventive treatment of latent tuberculosis infection (LTBI) has gradually gained a vital role in tuberculosis (TB) control worldwide since the 1950s. Currently, the global strategy in the treatment of TB is divided into two basic parts: in areas with a high incidence of TB, the main goal is to treat the active cases, but in areas with a low incidence of TB, the goal also includes prophylactic treatment for LTBI. According to the World Health Organization (WHO), approximately 2–3 billion people in the world are latently infected with *Mycobacterium tuberculosis* (Mtb), and 5%–15% of these people will suffer from reactivation of TB during their lifetime.^1^ Therefore, the treatment of LTBI directly influences the future global prevention of TB infection. At present, the study of LTBI relies heavily on screening for high-risk populations and on treatment strategies for the disease.

## SCREENING FOR LATENT TUBERCULOSIS INFECTION

Currently, a golden standard for the diagnosis of the LTBI is lacking. Because the amount of *Mycobacterium tuberculosis* is small in LTBI patients, diagnosis of LTBI mainly depends on the immune reaction of the host rather than the bacteria itself. There are two currently available screening tests for LTBI: the tuberculin skin test (TST) and interferon-γ release assays (IGRAs, including the QuantiFERON-TB Gold and the T-SPOT.TB test). As the conventional method for the diagnosis of LTBI, TST showed a high sensitivity in persons with normal immune responses^2^ and a sensitivity of 70% in human immunodeficiency virus (HIV)-infected person.^3^ However, TB vaccination (*Mycobacterium bovis* bacilli Calmette-Guérin, BCG) and exposure to non-tuberculous mycobacteria could cause cross-activity with the TST test, resulting in a low specificity.^4^ Compared to the TST, IGRAs reported a higher specificity in low-TB-prevalence areas and less cross-activity with the BCG vaccine in non-HIV-infected persons.^5,6^ However, in individuals infected with HIV, no difference was found in the diagnostic performance of tests for LTBI,^7^ although IGRAs were proven to be more cost-effective.^8^

Reactivation of LTBI accounts for a large proportion of active TB incidence, especially in countries with a low TB prevalence.^9,10^ Therefore, the predictive value for the development of active TB of IGRAs and the TST is very important and should be fully assessed. So to date, two meta-analyses have been conducted, and both reported little value for the prediction of active TB with either method.^11,12^ In fact, the majority of TST or IGRA-positive LTBI patients remain unreactivated after latent infection, and the TB risk was not significantly different between the two groups.^11,13^ A screening method with a better predictive value for ATB is needed in the future.

## RISK FACTORS FOR TUBERCULOSIS REACTIVATION

Only 5%–10% of screen-test-positive patients will develop active TB in the future.^10^ If prophylaxis is provided for all LTBI patients, it will result in an enormous waste of resources and increase the likelihood of anti-TB drug resistance. Some factors increase the risk of TB reactivation and require screening and treatment for LTBI. Table 1 lists reported risk factors and their relative risk of active TB.

### High-risk factors

#### HIV/AIDS

Approximately 1/4 of HIV deaths are caused by TB infection.^14^ Various studies have reported that HIV infection might lead to a 10–110 times higher risk of LTBI reactivation.^4,9,14^ A meta-analysis in 2010 reported that all LTBI prophylactic regimens would reduce the TB risks of HIV patients who were TST positive, whereas no evidence of efficacy was found among tuberculin skin-test-negative patients.^39^ However, in resource-constrained settings, full implementation of the TST or IGRAs has met with many difficulties. Therefore, the WHO recommended that all HIV patients who have unknown or positive screening test results and have no evidence of active TB receive prophylaxis, although patients with a positive TST or IGRA result might benefit more from preventive therapy. For HIV patients with negative screening test results, physicians should evaluate their individual TB risks and decide whether treatment should be prescribed.^40^ In 2015, the WHO's guidelines on latent TB again stressed the importance of LTBI treatment in HIV patients in both low- and high-income countries.^41^

#### Transplantation with immunosuppressant use

Patients who undergo organ transplantation are more susceptible to infections due to the use of immunosuppressive drugs. A study in Spain reported that kidney-, liver- and heart-transplant recipients had a TB incidence of 0.8%, 20 times higher than that of the general population, and no difference in TB risk was found among three types of transplantation.^16^ Retrospective studies reported a 0.65%–0.8% annual TB incidence rate after renal allografts in the United States, compared to 0.013 in the general population.^42,43^ Another study conducted in India reported a TB incidence of 11.8% among kidney-transplant recipients, 70 times higher than that of the general population.^17^ It would seem that the TB risk post-transplantation would be higher in third-world countries, but nevertheless, all studies recommended careful pre- and post-transplant examination for TB and LTBI. The WHO now recommends high- or middle-income countries with a low TB incidence rate (<100 per 100,000 population) to test and treat for LTBI in patients preparing for organ/hematologic transplantation.^41^

#### Silicosis

The relationship between silicosis and TB has long been recognized. Studies have reported that 25%–30% of silicosis patients develop TB,^23,44^ and the relative risk for TB reached 2.8 in silicosis patients compared to the general population.^23^ One study showed that preventive therapy could reduce the TB incidence rate by 12%–17% compared to the placebo group,^44^ and the WHO now recommends both testing and preventive treatment for LTBI for silicosis patients in high- or middle-income countries with a low TB incidence rate (<100 per 100,000 population).^41^ For countries with limited resources, whether to treat LTBI in silicosis patients remain to be discussed.

#### Close contact with pulmonary tuberculosis patients

People who have been recently infected with *Mtb* have a high risk of reactivation, and those who are close contacts of people with active TB have a high possibility of having been infected within the past 2 years.^3^ Studies have reported that the reactivation rate of TB is 15 times greater for those who have been recently infected (<two years).^4,15^ The American Thoracic Society (ATS) recommends that household contacts of TB patients with drug-susceptible TB and who are TST test positive undergo preventive treatment,^10^ whereas for close contacts of those with multidrug-resistant TB (MDR-TB), individual regimens based on drug susceptibility should be considered.^41^

#### Tumor necrosis factor-alpha blockers

Tumor necrosis factor-alpha (TNF-α) plays a key role in the body's inflammatory responses, and 5 TNF-α antagonists are currently used in the clinical fields (etanercept, adalimumab, infliximab, golimumab and certolizumab pegol). Randomized clinical trials (RCTs) on infliximab first reported a fourfold increase in the risk of TB infection,^45,46^ and soon, more studies reported a higher risk of TB in patients using TNF-α antagonists comparing to the placebo group, with a relative risk ranging from 1.6 to 25.1.^22^ In recent years, registry and longitudinal cohort studies have showed that the TB risk caused by the monoclonal antibody is generally higher than that of the receptor antibody.^47,48^ A meta-analysis of the published registry and longitudinal cohort studies found that the TB risks of infliximab and adalimumab were 2.78 and 3.88 times higher than that of etanercept, respectively.^49^ The WHO now recommends testing and treating for LTBI in all patients who plan to receive anti-TNF treatment in countries with a low TB risk.^41^

#### Chronic renal failure and hemodialysis

In the 1970s–1980s, many regions in the world reported a 10- to 12-fold increase of TB risk in patients with chronic renal failure (CRF) undergoing hemodialysis compared to the general population.^18,19,20^ Later, more studies confirmed a 6.9- to 52.5-fold increase of TB risk in dialysis patients.^21^ Other than the high prevalence of TB in the dialysis population, the diagnosis of TB in CRF patients had proven difficult. The sensitivity of the TST can be reduced by 50% during CRF and hemodialysis,^50^ and the localization of TB in CRF patients is often extrapulmonary, mostly presenting as tuberculous peritonitis and lymphadenitis.^21^ Thus, LTBI or TB cannot be simply ruled out with a negative TST result in CRF patients, but rather, IGRA tests and more invasive investigations are recommended.^21^ Currently, in several guidelines and reports, testing and prophylaxis of LTBI in CRF patients are suggested.^41,50,51,52^

### Moderate risk factors

#### Fibronodular diseases on chest x-rays

In the 1970s, a study reported a 6- to 19-folds increase of TB risk in individuals who were found to have old inactive TB lesions on chest radiography but did not have adequate treatment.^24^ The International Union Against Tuberculosis (IUAT) trials showed a 65% reduction in TB incidence with 6 months of isoniazid (INH) therapy for individuals with fibrotic lesions, proving the necessity of prophylaxis in this group.^53^ However, due to the widespread treatment of TB since the 1950s, especially in developed countries, the percentage of untreated patients has declined significantly. A national survey in the United States and Canada reported that only 1.4% of LTBI patients had old, healed TB,^54^ and a continuous decline in this percentage is foreseeable. Moreover, 30%–80% of TB infections could experience self-cure in the disease progression, and persons with evidence of healed TB lesions (i.e., calcified solitary pulmonary nodules, calcified hilar lymph nodes and apical pleural capping) do not suffer increased risk for TB reactivation.^10^ Therefore, the risk of previous TB infection is gradually reduced. The ATS recommended that patients who had evidence of or previous TB infection and no history of treatment be screened and treated for LTBI, and if an x-ray suggests healed primary TB, the decision regarding LTBI treatment should depend on other risk factors.^10^

#### Immigrants from countries with a high TB prevalence

In developed countries with a low TB prevalence, immigrants from high-TB-burden countries are one of the risk groups for TB.^25,55,56^ Therefore, screening and treating for LTBI and TB are conducted in many developed countries for foreign-born individuals. A study of 31 member countries of the Organization for Economic Cooperation and Development found that whereas 86.2% (16/29) of the members screen immigrants for active TB, only 55.2% (16/29) screened for LTBI.^57^ Moreover, some countries used solely the TST or IGRAs to screen for LTBI, and some use a combination of two methods for screening.^57^ A study in the Netherlands comparing TST and IGRA results among immigrants showed no evidence that one method was superior to the other,^58^ but the UK reported superior cost-effectiveness in IGRAs.^59^ Considering that some developing countries would use BCG vaccines to prevent TB prevalence, IGRAs might be more encouraged for LTBI screening. The cutoff value for screening also varies in different regions. Britain screens individuals who come from countries with a TB risk higher than 40/100,000 per year,^60^ and Japan screens people from countries with a risk of 100/100,000 per year.^51^ In the future, better uniformity in the screening methods and screening cutoff values should be implemented.

#### Health-care workers

Health-care workers are often at higher risk for nosocomially acquired TB compared to those not working in a health-care setting,^26,61^ which would result in secondary hospital outbreaks if not properly treated. The risk factors might be malfunctioning air conditioning systems (allowing recirculation of contaminated air),^62^ doctors without adequate self-protection who are present at procedures such as bronchoscopy,^63^ the emergence of the HIV epidemic^64,65^ or the increasing number of travelers from TB-prevalent countries. The TST and IGRAs are currently used for LTBI screening, and the WHO recommends that both testing and treating for LTBI be considered in middle- and high-income countries with a low TB incidence rate.^41^

#### Prisoners, homeless persons, and drug users

LTBI is more common among prisoners, homeless persons and drug users because these groups are usually underserved.^66,68^ These populations are more likely to be coinfected with HIV and are more difficult to treat adherently. Moreover, imprisonment is an important risk factor for the spread of drug-resistant TB infection.^69^ Several studies have evaluated the efficacy of prophylaxis for these groups, and it is widely recommended that these groups be screened and treated for LTBI.^10,41^ However, the efficacy of different regimens remains to be studied.

### Low-risk factors

#### Diabetes mellitus

Diabetes mellitus (DM) is known to increase the TB risk in individuals, and several studies have reported that the relative risk ranged from 1.16 to 7.83.^27,28,29,30,31,32^ However, no strong evidence supporting LTBI prophylaxis is available, and the WHO does not currently recommend systematic testing for LTBI.^41^ The reasons for this might be that the risk of TB in DM is relatively low, and no large-sample RCTs have been conducted concerning the subject. However, the TB risk is closely related to the patient's glycemic control, and a study has shown that patients with poor disease control have an increased risk of TB reactivation.^70^ Therefore, whether to treat LTBI patients who have poor glycemic control remains to be studied.

#### Smoking

Tobacco smoking can alter the lung immune responses to *Mtb* and can therefore contribute to a higher susceptibility to individual TB infection.^33,71^ The relative risk of TB infection in tobacco smokers compared to nonsmokers ranges from 2 to 3.4, and the TB reactivation and mortality rates are also higher in the tobacco group.^33,34,35^ For decades, physicians have debated whether LTBI patients exposed to tobacco smoking should receive prophylaxis, but no recommendation has been made in the current strategy.^41^ The reasons include financial and health issues. In low- and middle-income nations, approximately 50% of men and 8% of women smoke, and if every LTBI patient exposed to tobacco is treated, the number of patients to treat would cause huge financial and medical waste.^72^ On the other hand, a study has estimated that the complete elimination of tobacco smoking would lead to a 14%–52% reduction in TB risk.^73^ Therefore, the current best and most efficient strategy might still be to promote antismoking campaigns worldwide.

#### Use of corticosteroids

For patients who are being treated with corticosteroids, the risk of TB reactivation increases 2.8- to 7.7-fold.^36^ Although there is a lack of evidence to support the preventive treatment of LTBI in all patients who are administered corticosteroids, it is still reasonable to evaluate the risks of TB in these patients. If a patient is prescribed a large dose of corticosteroids and has a high-risk for TB reactivation, such as HIV infection, silicosis and organ transplantation, prophylactic treatment might lower the incidence rate of TB.

#### Underweight status

Being underweight (≥10% deviation from ideal weight) can cause a 2- to 3-fold increase in active TB development compared to the general population.^37,38^ In their 2000 statement, the ATS held a vague position concerning whether underweight people should receive preventive treatment, despite regarding underweight status as a risk factor for TB development.^10^ The TBNET consensus statement also considered LTBI treatment unnecessary,^22^ and the WHO noted that the benefits of routine testing and treatment of LTBI for underweight persons were nonsignificant. The current recommendation states that testing and treatment of LTBI should be conducted only when underweight status is accompanied by any of the high-risk factors.^41^

## PREVENTIVE TREATMENT OF LATENT TUBERCULOSIS INFECTION IN NON-HIV PATIENTS

The preventive treatment of latent TB has improved greatly in recent decades. The treatment of high-risk LTBI populations has been proven effective by many clinical trials in reducing the recurrence rate of active TB. Table 2 lists current prophylactic therapies and their dosages, as recommended by the WHO.

### Isoniazid monotherapy

Isoniazid monotherapy was the first experimental therapy for the preventive treatment of LTBI. Between the 1950s and 1970s, many randomized clinical studies were launched on isoniazid monotherapy, with regimens ranging from 3 months of isoniazid (3INH) therapy to 12 months of isoniazid (12INH) therapy, and all the results strongly suggested that daily or intermittent isoniazid might reduce the incidence of TB reactivation.^39,53,74^ The largest trial ever conducted was by the IUAT, in which approximately 28,000 TST-positive persons with fibrotic lesions were enrolled. The study reported that compared with placebo, the 3INH, 6 months of isoniazid (6INH) and 12INH regimens reduced the TB risk by 21%, 65% and 75%, respectively, within 5 years of follow-up. Both 6INH and 12INH therapies showed a more significant reduction in TB incidence than the placebo group and the 3INH group; however, no statistical significance was observed between the 6INH and 12INH regimens.^53^ In 1999, based on the United States Public Health Service trials conducted in the 1950s and 1960s, a secondary modeling reanalysis reported that daily 9-month isoniazid (9INH) therapy might achieve the maximum efficacy.^75^ In a recently published trial of isoniazid preventive therapy in South African gold miners, the results showed a reduction of TB risk during 9INH treatment compared to the control group (incidence rate ratio, 0.42; 95% CI, 0.20–0.88), but the protection was lost after 2 years of follow-up.^76^ This result suggested a higher TB reactivation rate in high TB-prevalence areas despite prophylaxis. Currently, the WHO recommends both 6INH and 9INH regimens as equivalent options, and no significant difference in efficacy has been found between the two regimens.^41^

With the widespread use of isoniazid preventive treatment for latent TB, side effects have gradually become a concern. In 1970–1971, the United States Public Health Center examined 14,000 patients who were administered isoniazid, and reported that the occurrence of hepatitis was 1–2.3%, and the risk increased for patients with a history of chronic liver disease or alcohol intake.^10^ In 2008, the 9INH regimen was reported to cause severe liver toxicity in 3.8% of the patients, and the compliance rate varied greatly among different studies.^77,78^ Other adverse effects of isoniazid monotherapy, such as peripheral neuropathy, have also been noted.

### Rifampicin-containing therapies

Silicosis is a high-risk factor for TB. In 1992, the Hong Kong Thoracic Society and the British Medical Research Council conducted a randomized controlled clinical trial targeting Chinese silicosis patients. The researchers compared the TB incidence rate among the 3 months of rifampicin plus isoniazid regimen (3RIF + INH), 3 months of rifampicin regimen (3RIF), 6INH and the placebo group. The study found that the 5-year cumulative incidence rate of active TB in the placebo group was higher than in the other groups (placebo: 27%, 3RIF + INH group: 16%, 6INH group: 14%, 3RIF group: 10%).^44^ This clinical study was the first to support rifampicin monotherapy and the 3RIF + INH regimen as the treatment for LTBI. Later on, more studies were conducted on rifampicin-containing therapies. Although no trial showed that the rifampicin-containing regimens had a significantly better prophylactic result than the INH regimens, studies found that the 4RIF regimen had less liver toxicity and was more cost effective.^77,79^ In 2000, the ATS recommended 4RIF as an alternative to 9INH,^10^ and the British Thoracic Society recommended 3RIF + INH as an alternative to 6INH.^80^

### High-dosage rifapentine plus isoniazid therapy

A random, unblinded, noninferiority study conducted from 2001 to 2008 reported that 3 months of weekly rifapentine plus isoniazid therapy (3RPT + INH) did not have a disadvantage compared with 9INH therapy in non-HIV patients (the cumulative incidence rates of active TB were 0.19% and 0.43%, respectively) and had significantly lower liver toxicity (OR 0.16, 95% CI: 0.1–0.27).^81^ Another recent study reported systemic drug reactions, mostly flu-like syndromes, among persons (3.5%) receiving the 3RPT + INH regimen.^82^ The advantage of 3RPT + INH is clear, characterized by a short treatment course, reduction of the frequency of medication and fewer hepatotoxicity events. In the 2015 WHO guidelines, the 3RPT + INH regimen is recommended as a treatment option equivalent to the 6INH and 9INH regimens, but the quality of the evidence is only moderate to low.^41^ To date, treatment in the 3RPT + INH group was directly observed in clinics, and therefore, the treatment efficacy of a self-administered 3RPT + INH regimen remains to be studied.

### Rifampicin plus pyrazinamide therapy

Two-month rifampicin plus pyrazinamide (2RZ) regimen was first proved effective in clinical studies and was recommended as an alternative treatment to isoniazid.^83,84^ However, studies soon reported that the 2RZ regimen could cause serious liver toxicity,^85,86^ which in severe cases could lead to death. These reports evoked vigilance, and in 2003, the ATS/CDC recommended against this regimen in general. The 2RZ regimen should be provided to selected patients only when other alternative regimens cannot be completed and only with the consultation and oversight of physicians.^87^

### Comparison between regimens

Currently, the 6INH and 9INH regimens are the classic recommended regimens for LTBI treatment. Although the 3RPT + INH, 3-4RIF + INH and 3-4RIF regimens are also recommended by the WHO, none of these regimens has shown superiority over isoniazid monotherapy. In some studies, the 3-4RIF and 3RPT + INH regimens were reported to have fewer hepatotoxicity events, but the quality of evidence supporting this is only moderate to low.^41^ Therefore, for non-HIV patients, the first-line choice should still be the 6 or 9INH regimen, and the treatment efficacy and safety of 3RPT + INH and 3–4 RIF should be further studied.

## PREVENTIVE THERAPY FOR TARGETED GROUPS WITH HIGH-RISK FACTORS

### HIV-infected patients

Several clinical studies showed that isoniazid monotherapy, with a regimen ranging from 6 to 12 months, could reduce the probability of TB reactivation by 32–67% in HIV-infected LTBI patients.^88,89,90,91^ However, in high TB-prevalence regions, the reactivation rate of ATB would be higher.^92^ Continuous isoniazid monotherapy was also explored for its potential benefit in settings with a high HIV and TB prevalence. One large, RCT reported that 36 months of isoniazid therapy (36INH) showed a superior efficacy than 6INH in LTBI treatment,^93^ whereas another study showed that continuous isoniazid therapy up to 6 years had no superiority over 6INH but more adverse reactions.^94^ The efficacy between multidrug regimens was also compared. The results showed that the 3RPT + INH and 3RIF + INH (daily or twice weekly) regimens both reduced the TB risk in HIV-infected LTBI patients, although no significant difference in treatment efficacy was observed compared to the 6INH regimen.^90,94^ Additionally, side effects were more likely to take place with multidrug therapies.^90^ Currently, the WHO strongly recommends at least 6 months of isoniazid preventive therapy (6INH, 9INH, 12INH) for HIV-infected patients and suggests a continuous 36INH regimen as the surrogate treatment, especially in regions with high HIV and TB prevalence.^40^

### Silicosis patients

For silicosis patients, most of the data have come from the Hong Kong Chest Service. In a 5-year follow-up, the 3RIF regimen was considered to have the best efficacy when compared to the placebo group, reducing the TB risk by 17%. Both the 6INH and 3RIF + INH regimens also reduced the TB risk in silicosis patients (14% and 12%, respectively), and no significant differences were observed among the three prophylactic regimens.^44^ Because 3RIF has the least hepatotoxicity among the three regimens,^41^ rifampicin monotherapy might be the first choice for the preventive treatment in silicosis patients, although further studies are required.

### Organ-transplantation recipients with immunosuppressant use

Various studies have reported the prophylactic value of different isoniazid monotherapy (e.g., 6INH and 12INH) in post-kidney-transplant recipients,^95,96^ all in high-TB-prevalence areas (India, Brazil and Pakistan). Systematic reviews showed that isoniazid prophylaxis could significantly reduce the post-kidney-transplant TB risk by 65%–69% in recipients who were at risk of TB reactivation, but hepatotoxicity risks were also reported.^97,98^ We recommend isoniazid monotherapy as the prophylactic regimen in transplantation recipients, but hepatotoxicity events should be carefully monitored in the future.

### TNF-α antagonist recipients

A meta-analysis was conducted to evaluate the efficacy of preventive treatment, and the results showed that the TB risk was decreased by 65% (RR = 0.35, P = 0.02) in patients receiving prophylaxis compared to those who did not.^18^ However, the studies enrolled mostly rheumatoid arthritis patients, and the regimens differed among the included studies (e.g., 6INH, 9INH, 3INH + RIF).^99,100,101,102^ One study reported a 97% decrease in TB risk using 9INH, whereas another study reported a 33% risk decrease using 6INH or 3INH + RIF,^99,101^ suggesting that the 9INH regimen might be more effective in treating LTBI. However, currently, no RCT or cohort directly comparing the efficacy among different regimens is available.

### Close contacts of pulmonary tuberculosis patients

The WHO, the ATS and the British Thoracic Society all recommend screening and treatment for LTBI for close contacts of TB patients with drug-susceptible TB.^10,41,80^ However, for close contacts of MDR-TB, controversy remains regarding the efficacy and necessity of prophylaxis for LTBI. Because of the limited studies on preventive treatment for contacts of MDR-TB, systematic reviews all noted that high-quality evidence to support the feasibility and safety of prophylactic treatment is still lacking.^103,104^ Additionally, the regimens for LTBI patients exposed to MDR-TB are not clear, and some studies have recommended that individual regimens be based on drug susceptibility.^41^ In a prospective study published in 2014, a 12-month fluoroquinolone regimen was administered to 119 contacts of MDR-TB patients, and none of the 104 contacts who received the treatment developed MDR-TB, while 3 of the 15 contacts who refused the treatment developed the disease.^105^ This study suggested that treatment for contacts of MDR-TB might prevent MDR-TB development, but further research is urgently needed.

### Chronic renal failure and hemodialysis

One study in India reported a 60% reduction in the TB risk in CRF patients undergoing hemodialysis when treated with 12INH, indicating the efficacy of prophylaxis. However, hepatitis developed in 16.7% of the patients, and most of them were hepatitis B or C positive. These results indicated that patients with previous liver diseases have a higher risk of liver damage during isoniazid prophylaxis.^106^ Currently, no worldwide consensus has been reached concerning treatment options. The ATS recommended the 9INH regimen (accompanied by pyridoxine) to treat for LTBI in CRF patients undergoing hemodialysis,^10,21^ and the British Thoracic Society recommended three other potential regimens: the 6INH, 3RIF + INH and 4-6RIF regimens.^50^ Both recommendations have little evidence, and further studies are strongly required.

## CONCLUSION

The prophylaxis of LTBI plays an important role in the prevention and treatment of TB. IGRAs and the TST are both used to screen for LTBI, and although some studies in low-TB-prevalence areas reported a higher specificity with IGRAs than with the TST, neither method had a satisfying predictive value for active TB. In the future, a screening method with a better predictive value should be explored. High-risk factors (HIV/AIDs, transplantation, silicosis, TNF-α blockers, close contacts, kidney dialysis) contribute to a significantly increased TB reactivation rate, and for countries with a low TB prevalence, patients with high-risk factors should undergo screening and treatment for LTBI.

At present, the WHO recommends five prophylactic regimens—6INH, 9INH, 3-4RIF, 3-4RIF + INH and 3RPT + INH—none of which has shown superiority over the conventional 6INH or 9INH therapies. The 3-4RIF and 3RPT + INH regimens have been reported to have fewer hepatotoxicity events, but the quality of evidence is low. Further research regarding the treatment efficacy and safety of the 3RPT + INH and 3–4 RIF regimens is required. For high-risk groups, isoniazid monotherapy could reduce the TB risk in HIV-infected patients and transplant recipients, but for others, little evidence is available to draw a conclusion at this time. In the future, high-risk population screening and new preventive treatment therapies for specific target groups and the drug resistance that follows will be the keys to improve the prophylaxis of latent TB.

## Figures and Tables

**Table 1:** Risk factors for TB activation

			**WHO's recommendation for screening and treatment for LTBI^41^**	
**Risk factor**	**TB risk^a^**	**Reference(s)**	**Country A^b^**	**Country B^c^**
High-risk factors				
HIV/AIDS	10–100	Landry *et al*.,^4^ Hourburgh *et al*.^9^ and WHO^14^	Required	Required
Close contacts	15	Landry *et al*.^4^ and Sutherland *et al*.^15^	Required	Required for close contacts (<5 years old)
Organ-transplantation recipients	20–70	Aguado *et al*.^16^ and Sakhuja *et al*.^17^	Required	Not mentioned
Chronic renal failure requiring dialysis	6.9–52.5	Andrew *et al*.,^18^ Lundin *et al*.,^19^Belcon *et al*.^20^ and Hussein *et al*.^21^	Required	Not mentioned
TNF-alpha blockers	1.6–25.1	Solovic *et al*.^22^	Required	Not mentioned
Silicosis	2.8	Cowie *et al*.^23^	Required	Not mentioned
Moderate-risk factors				
Fibronodular disease on chest x-ray	6–19	Grzybowski *et al*.^24^	Not mentioned	Not mentioned
Immigrants from high-TB-prevalence countries	2.9–5.3	Baussano *et al*.^25^	Options to be considered	Not mentioned
Health-care workers	2.55	Chu *et al*.^26^	Options to be considered	Not mentioned
Prisoners, homeless persons, illicit drug users	–	–	Options to be considered	Not mentioned
Low-risk factors				
Diabetes mellitus	1.6–7.83	Harries *et al*.,^27^ Dobler *et al*.,^28^ Jeon *et al*.,^29^ Boucot *et al*.,^30^ Kim *et al*.^31^ and Baker *et al*.^32^	Not recommended	Not mentioned
Smoking	2–3.4	Altet *et al*.,^33^ Slama *et al*.^34^ and Maurya *et al*.^35^	Not recommended	Not mentioned
Use of corticosteroids	2.8–7.7	Jick *et al*.^36^	Not recommended	Not mentioned
Underweight	2–3	Palmer *et al*.^37^ and Comstock *et al*.^38^	Not recommended	Not mentioned

a Relative risk of TB compared to the general population.

b In high- and upper-middle-income countries with an estimated TB incidence less than 100/100,000 population.

c For resource-limited countries and other middle-income countries that do not belong to country A.

**Table 2:** WHO-recommended preventive regimens for latent tuberculosis infection^41^

Regimen*	**Dosage**	**HepatotoxicityOR (95% CI)**	**Treatment efficacy**	
6INH	Children: 10 mg/kg/d Adults: 5 mg/kg/d Maximum dose: 300 mg		Compared to placebo: 0.99 (0.42–2.32)	Equivalent to 9INH and 3RPT + INH regimens
9INH	Children: 10 mg/kg/d Adults: 5 mg/kg/d Maximum dose: 300 mg		–	Equivalent to 6INH and 3RPT + INH regimens
3-4RIF	Children: 10 mg/kg/d Adults: 10 mg/kg/d Maximum dose: 600 mg		Compared to 6INH: 0.03 (0.00–0.48)	Maybe equivalent to 6INH regimen
3-4RIF + INH	Rifampicin: Children: 10 mg/kg/d Adults: 10 mg/kg/d Maximum dose: 600 mg	Isoniazid:Children: 10 mg/kg/d Adults: 5 mg/kg/d Maximum dose: 300 mg	Compared to 6INH: 0.89 (0.52–1.55)	Maybe equivalent to 6INH regimen
3RPT + INH	Rifapentine: 10.0–14.0 kg: 300 mg 14.1–25.0 kg: 450 mg 25.1–32.0 kg: 600 mg 32.1–49.9 kg: 750 mg Maximum dose: 900 mg	Isoniazid:Children: 15 mg/kg/d Adults: 15 mg/kg/d Maximum dose: 900 mg	Compared to 6INH: 1.0 (0.50–1.99)Compared to 6INH: 0.16 (0.10–0.27)	Equivalent to 6INH and 9INH regimens

* Regimen: 6INH: daily isoniazid for 6 months; 9INH: daily isoniazid for 9 months; 3-4RIF: daily rifampicin for 3–4 months; 3–4RIF + INH: daily rifampicin plus isoniazid for 3–4 months; 3RPT + INH: weekly rifapentine plus isoniazid for 3 months.

## References

[bib1] World Health Organization. *Global Tuberculosis Report 2015*. Geneva: WHO, 2015. Available at http://www.who.int/tb/publications/global_report/en/(accessed 23 November 2015).

[bib2] Rose DN, Schecter CB, Adler JJ. Interpretation of the tuberculin skin test. J Gen Intern Med 1995; 10: 635–642. 8583267 10.1007/BF02602749

[bib3] Parekh MJ, Schluger NW. Treatment of latent tuberculosis infection. Ther Adv Respir Dis 2013; 7: 351–356. 24056289 10.1177/1753465813503028

[bib4] Landry J, Menzies D. Preventive chemotherapy. Where has it got us? Where to go next? Int J Tuberc Lung Dis 2008; 12: 1352–1364. 19017442

[bib5] Menzies D, Pai M, Comstock G. Meta-analysis: new tests for the diagnosis of latent tuberculosis infection: areas of uncertainty and recommendations for research. Ann Intern Med 2007; 146: 340–354. 17339619 10.7326/0003-4819-146-5-200703060-00006

[bib6] Pai M, Riley LW, Colford JMJr. Interferon-gamma assays in the immunodiagnosis of tuberculosis: a systematic review. Lancet Infect Dis 2004; 4: 761–776. 15567126 10.1016/S1473-3099(04)01206-X

[bib7] Cattamanchi A, Smith R, Steingart KR et al. Interferon-gamma release assays for the diagnosis of latent tuberculosis infection in HIV-infected individuals: a systematic review and meta-analysis. J Acquir Immune Defic Syndr 2011; 56: 230–238. 21239993 10.1097/QAI.0b013e31820b07ab PMC3383328

[bib8] Linas BP, Wong AY , Freedberg KA . Priorities for screening and treatment of latent tuberculosis infection in the United States. Am J Respir Crit Care Med 2011; 184: 590–596. 21562129 10.1164/rccm.201101-0181OC PMC3175546

[bib9] Horsburgh CR, Rubins EJ. Latent tuberculosis infection in the United States. N Engl J Med 2011; 364: 1441–1448. 21488766 10.1056/NEJMcp1005750

[bib10] American Thoracic Society. Targeted tuberculin testing and treatment of latent tuberculosis infection. MMWR Recomm Rep 2000; 49: 1–51. 10881762

[bib11] Rangaka MX, Wilkinson KA, Glynn JR et al. Predictive value of interferon-γ release assays for incident active tuberculosis: a systematic review and meta-analysis. Lancet Infect Dis 2012; 12: 45–55. 21846592 10.1016/S1473-3099(11)70210-9 PMC3568693

[bib12] Metcalfe JZ, Everett CK, Steingart KR et al. Interferon-γ release assays for active pulmonary tuberculosis diagnosis in adults in low- and middle-income countries: systematic review and meta-analysis. J Infect Dis 2011; 204: S1120–S1129. 21996694 10.1093/infdis/jir410 PMC3192542

[bib13] Watkins RE, Brennan R, Plant AJ. Tuberculin reactivity and the risk of tuberculosis: a review. Int J Tuberc Lung Dis 2000; 4: 895–903. 11055755

[bib14] World Health Organization. Global tuberculosis control: a short update to the 2009 Report. Geneva: WHO, 2010. http://www.who.int/tb/features_archive/globalreport09_update_8dec09/en/ (accessed 9 September 2015).

[bib15] Sutherland I. Recent studies in the epidemiology of tuberculosis, based on the risk of being infected with tubercle bacilli. Adv Tuberc Res 1976; 19: 1–63. 823803

[bib16] Aguado JM, Herrero JA, Gavalda J et al. Clinical presentation and outcome of tuberculosis in kidney, liver and heart transplant recipients in Spain. Spanish Transplantation Infection Study Group, GESITRA. Transplantation 1997; 63: 1278–1286. 9158022 10.1097/00007890-199705150-00015

[bib17] Sakhuja V, Jha V, Varma PP et al. The high incidence of tuberculosis among renal transplant recipients in India. Transplantation 1996; 61: 211–215. 8600625 10.1097/00007890-199601270-00008

[bib18] Andrew OT, Schoenfeld PY, Hopewell PC et al. Tuberculosis in patients with end-stage renal disease. Am J Med 1980; 68: 59–65. 7350806 10.1016/0002-9343(80)90166-7

[bib19] Lundin AP, Adler AJ, Berlyne GM et al. Tuberculosis in patients undergoing maintenance hemodialysis. Am J Med 1979; 67: 597–602. 495629 10.1016/0002-9343(79)90240-7

[bib20] Belcon MC, Smith EK, Kahana LM et al. Tuberculosis in dialysis patients. Clin Nephrol 1982; 17: 14–18. 7055993

[bib21] Hussein MM, Mooij JM, Roujouleh H. Tuberculosis and chronic renal disease. Semin Dial 2003; 16: 38–44. 12535299 10.1046/j.1525-139x.2003.03010.x

[bib22] Solovic I, Sester M, Gomez-Reino JJ et al. The risk of tuberculosis related to tumour necrosis factor antagonist therapies: a TBNET Consensus Statement. Eur Respir J 2010; 36: 1185–1206. 20530046 10.1183/09031936.00028510

[bib23] Cowie RL. The epidemiology of tuberculosis in gold miners with silicosis. Am J Respir Crit Care Med 1994; 150: 1460–1462. 7952577 10.1164/ajrccm.150.5.7952577

[bib24] Grzybowski S, Fishaut H, Rowe J et al. Tuberculosis among patients with various radiologic abnormalities, followed by the chest clinic service. Am Rev Respir Dis 1971; 104: 605–608. 5094062 10.1164/arrd.1971.104.4.605

[bib25] Baussano I, Mercadante S, Pareek M et al. High rates of *Mycobacterium tuberculosis* among socially marginalized immigrants in low-incidence area, 1991–2010, Italy. Emerg Infect Dis 2013; 19: 1437–1445. 23965807 10.3201/eid1909.120200 PMC3810899

[bib26] Chu H, Shih CJ, Lee YJ et al. Risk of tuberculosis among healthcare workers in an intermediate-burden country: a nationwide population study. J Infect 2014; 69: 525–532. 25135230 10.1016/j.jinf.2014.06.019

[bib27] Harries AD, Lin Y, Satyanarayana S et al. The looming epidemic of diabetes-associated tuberculosis: learning lessons from HIV-associated tuberculosis. Int J Tuberc Lung Dis 2011; 15: 1436–1444. 21902876 10.5588/ijtld.11.0503

[bib28] Dobler CC, Flack JR, Marks GB. Risk of tuberculosis among people with diabetes mellitus: an Australian nationwide cohort study. BMJ Open 2012; 2: e000666. 10.1136/bmjopen-2011-000666 PMC3282295 22331390

[bib29] Jeon CY, Murray MB. Diabetes mellitus increases the risk of active tuberculosis: a systematic review of 13 observational studies. PLoS Med 2008; 5: e152. 18630984 10.1371/journal.pmed.0050152 PMC2459204

[bib30] Boucot KR. Diabetes mellitus and pulmonary tuberculosis. J Chronic Dis 1957; 6: 256–279. 13463061 10.1016/0021-9681(57)90007-3

[bib31] Kim SJ, Hong YP, Lew WJ et al. Incidence of pulmonary tuberculosis among diabetics. Tubercle Lung Dis 1995; 76: 529–533. 10.1016/0962-8479(95)90529-4 8593374

[bib32] Baker MA, Lin HH, Chang HY et al. The risk of tuberculosis disease among persons with diabetes mellitus: a prospective cohort study. Clin Infect Dis 2012; 54: 818–825. 22238171 10.1093/cid/cir939

[bib33] Altet MN, Alcaide J, Plans P et al. Passive smoking and risk of pulmonary tuberculosis in children immediately following infection. A case control study. Tubercle Lung Dis 1996; 77: 537–544. 10.1016/s0962-8479(96)90052-0 9039447

[bib34] Slama K, Chiang CY, Enarson DA et al. Tobacco and tuberculosis: a qualitative systematic review and meta- analysis. Int J Tuberc Lung Dis 2007; 11: 1049–1061. 17945060

[bib35] Maurya V, Vijayan VK, Shah A. Smoking and tuberculosis: an association overlooked. Int J Tuberc Lung Dis 2002; 6: 942–951. 12475139

[bib36] Jick SS, Lieberman ES, Rahman MU et al. Glucocorticoid use, other associated factors, and the risk of tuberculosis. Arthritis Rheum 2006; 55: 19–26. 16463407 10.1002/art.21705

[bib37] Palmer CE, Jablon S, Edwards PQ. Tuberculosis morbidity of young men in relation to tuberculin sensitivity and body build. Am Rev Tuberc 1957; 76: 517–539. 13470303 10.1164/artpd.1957.76.4.517

[bib38] Comstock GW. Frost revisited: the modern epidemiology of tuberculosis. Am J Epidemiol 1975; 101: 363–382. 1093397 10.1093/oxfordjournals.aje.a112105

[bib39] Akolo C, Adetifa I, Shepperd S et al. Treatment of latent tuberculosis infection in HIV Infected Persons. Cochrane Database Syst Rev 2010; CD000171. doi: 10.1002/14651858. 10.1002/14651858.CD000171.pub3 PMC7043303 20091503

[bib40] World Health Organization. Guidelines for intensified tuberculosis case-finding and isoniazid preventive therapy for people living with HIV in resource-constrained settings. Geneva: WHO, 2011. Available at http://www.who.int/HIV/pub/tb/9789241500708/en/ (accessed 17 October 2015).

[bib41] World Health Organization. *Guidelines on the management of latent tuberculosis infection*. Geneva: WHO, 2015. Available at http://www.who.int/tb/publications/ltbi_document_page/en/ (accessed 23 October 2015). 25973515

[bib42] Lloveras J, Peterson PK, Simmons RL et al. Mycobacterial infections in renal transplant patients. Arch Intern Med 1982; 142: 888–892. 7044331

[bib43] Lichtenstein IH, MacGregor RR. Mycobacterial infections in renal transplant patients: report of 5 cases and review of literature. Rev Infect Dis 1983; 5: 216–226. 6342092 10.1093/clinids/5.2.216

[bib44] Hong Kong Chest Service/Tuberculosis Research Centre, Madras/British Medical Research Council. A double-blind placebo-controlled clinical trial of three antituberculosis chemoprophylaxis regimens in patients with silicosis in Hong Kong. Am Rev Respir Dis 1992; 145: 36–41. 1731596 10.1164/ajrccm/145.1.36

[bib45] Maini R, St Clair EW, Breedveld F et al. Infliximab (chimeric anti-tumour necrosis factor alpha monoclonal antibody) versus placebo in rheumatoid arthritis patients receiving concomitant methotrexate: a randomised phase III trial. ATTRACT Study Group. Lancet 1999; 354: 1932–1939. 10622295 10.1016/s0140-6736(99)05246-0

[bib46] Keane J, Gershon S, Wise RP et al. Tuberculosis associated with infliximab, a tumor necrosis factor alpha-neutralizing agent. N Engl J Med 2001; 345: 1098–1104. 11596589 10.1056/NEJMoa011110

[bib47] Fonseca JE, Canhao H, Silva C et al. Tuberculosis in rheumatic patients treated with tumour necrosis factor alpha antagonists: the Portuguese experience. Acta Reumatol Port 2006; 31: 247–253. 17094336

[bib48] Brassard P, Kezouh A, Suissa S. Antirheumatic drugs and the risk of tuberculosis. Clin Infect Dis 2006; 43: 717–722. 16912945 10.1086/506935

[bib49] Ai JW, Zhang S, Ruan QL et al. The Risk of Tuberculosis in Patients with Rheumatoid Arthritis Treated with Tumor Necrosis Factor-α Antagonist: A Metaanalysis of Both Randomized Controlled Trials and Registry/Cohort Studies. J Rheumatol 2015 Dec; 42: 2229–2237. doi: 10.3899/jrheum.150057. 26472414 10.3899/jrheum.150057

[bib50] British Thoracic Society Standards of Care Committee, Joint Tuberculosis Committee, Milburn H, et al. Guidelines for prevention and management of *Mycobacterium tuberculosis* infection and disease in adult patients with chronic kidney disease. Thorax 2010; 65: 557–570. 20522863 10.1136/thx.2009.133173

[bib51] Prevention Committee of the Japanese Society for Tuberculosis, Treatment Committee of the Japanese Society for Tuberculosis. Treatment Guidelines for Latent Tuberculosis Infection. Kekkaku 2014; 89: 21–37. 24654427

[bib52] Cengiz K. Should tuberculosis prophylaxis be given to chronically dialyzed patients? Nephron 2000; 86: 411–413. 11221690 10.1159/000045828

[bib53] International Union Against Tuberculosis Committee on prophylaxis. Efficacy of various durations of isoniazid preventive therapy for tuberculosis: five years of follow-up in the IUAT trial. Bull World Health Organ 1982; 60: 555–564. 6754120 PMC2536088

[bib54] Horsburgh CR, Goldberg S, Bethel J et al. Tuberculosis Epidemiologic Studies Consortium. Latent tuberculosis infection treatment acceptance and completion in 2002 in the United States and Canada. Chest 2010; 137: 401–409. 19793865 10.1378/chest.09-0394

[bib55] Blum RN, Polish LB, Tapy JM et al. Results of screening for tuberculosis in foreign-born persons applying for adjustment of immigration status. Chest 1993; 103: 1670–1674. 8404083 10.1378/chest.103.6.1670

[bib56] Cain KP, Benoit SR, Winston CA et al. Tuberculosis among foreign-born persons in the United States. JAMA 2008; 300: 405–412. 18647983 10.1001/jama.300.4.405

[bib57] Pareek M, Baussano I, Abudakar I et al. Evaluation of immigrant. Emerg Infect Dis 2012; 18: 1422–1429. 22931959 10.3201/eid1809.120128 PMC3437731

[bib58] Kik SV, Franken WP, Mensen M et al. Predictive value for progression to tuberculosis by IGRA and TST in immigrant contacts. Eur Respir J 2010; 35: 1346–1353. 19840963 10.1183/09031936.00098509

[bib59] Pareek M, Bond M, Shorey J et al. Community-based evaluation of immigrant tuberculosis screening using interferon γ release assays and tuberculin skin testing: observational study and economic analysis. Thorax 2013; 68: 230–239. 22693179 10.1136/thoraxjnl-2011-201542 PMC5741173

[bib60] National Institute for Health and Clinical Excellence: NICE clinical guideline 117. Tuberculosis: clinical diagnosis and management of tuberculosis, and measures for its prevention and control. London, 2011. 22720337

[bib61] Hernández M, Casar C, García J et al. Latent tuberculosis infection screening in healthcare workers in four large hospitals in Santiago, Chile. Rev Chilena Infectol 2014; 31: 254–260. 25146198 10.4067/S0716-10182014000300002

[bib62] Catanzaro A. Nosocomial tuberculosis. Am Rev Respir Dis 1982; 125: 559–562. 7081816 10.1164/arrd.1982.125.5.559

[bib63] Malasky C, Jordan T, Potulski F et al. Occupational tuberculosis infections among pulmonary physicians in training. Am Rev Respir Dis 1990; 142: 505–507. 2389901 10.1164/ajrccm/142.3.505

[bib64] Nardell EA. Dodging droplet nuclei: reducing the probability of nosocomial tuberculosis transmission in the AIDS era. Am Rev Respir Dis 1990; 140: 501–503. 10.1164/ajrccm/142.3.501 2389900

[bib65] Stead WW. Annual tuberculosis screening of hospital employees-an idea whose time has not passed (Editorial). Am Rev Respir Dis 1987; 136: 803–804. 3662232 10.1164/ajrccm/136.4.803

[bib66] Graham NM, Nelson KE, Solomon L et al. Prevalence of tuberculin positivity and skin test anergy in HIV-1-seropositive and -seronegative intravenous drug users. JAMA 1992; 267: 369–372. 1727959

[bib67] Zolopa AR, Hahn JA, Gorter R et al. HIV and tuberculosis infection in San Francisco's homeless adults. JAMA 1994; 272: 455–461. 8040981

[bib68] Wallace R, Wallace D. Community marginalization and the diffusion of disease and disorder in the United States. BMJ 1997; 314: 1341–1345. 9158474 10.1136/bmj.314.7090.1341 PMC2126575

[bib69] Anderson C, Story A, Brown T et al. Tuberculosis in UK prisoners: a challenge for control. J Epidemiol Community Health 2010; 64: 373–376. 20231737 10.1136/jech.2009.094375

[bib70] Leung CC, Lam TH, Chan WM et al. Diabetic control and risk of tuberculosis: a cohort study. Am J Epidemiol 2008; 167: 1486–1494. 18400769 10.1093/aje/kwn075

[bib71] Chan ED, Kinney WH, Honda JR et al. Tobacco exposure and susceptibility to tuberculosis: is there a smoking gun? Tuberculosis 2014; 94: 544–550. 25305002 10.1016/j.tube.2014.08.010

[bib72] Chan ED, Keane J, Iseman MD. Should cigarette smoke exposure be a criterion to treat latent tuberculous infection? Am J Respir Crit Care Med 2010; 182: 990–992. 20947905 10.1164/rccm.201006-0861ED

[bib73] van Zyl Smit RN, Pai M, Yew WW et al. Global lung health: the colliding epidemics of tuberculosis, tobacco smoking, HIV and COPD. Eur Respir J 2010; 35: 27–33. 20044459 10.1183/09031936.00072909 PMC5454527

[bib74] Egsmose T, Ang'awa JO, Poti SJ. The use of isoniazid among household contacts of open cases of pulmonary tuberculosis. Bull World Health Organ 1965; 33: 419–433. 5321762 PMC2475854

[bib75] Comstock GW. How much isoniazid is needed for prevention of tuberculosis among immunocompetent adults? Int J Tuberc Lung Dis 1999; 3: 847–850. 10524579

[bib76] Churchyard GJ, Fielding KL, Lewis JJ et al. A trial of mass isoniazid preventive therapy for tuberculosis control. N Engl J Med 2014; 370: 301–310. 24450889 10.1056/NEJMoa1214289

[bib77] Menzies D, Dion MJ, Rabinovitch B et al. Treatment completion and costs of a randomized trial of rifampin for 4 months versus isoniazid for 9 months. Am J Respir Crit Care Med 2004; 170: 445–449. 15172892 10.1164/rccm.200404-478OC

[bib78] Menzies D, Long R, Trajman A et al. Adverse events with 4 months of rifampin therapy or 9 months of isoniazid therapy for latent tuberculosis infection: a randomized trial. Ann Intern Med 2008; 149: 689–697. 19017587 10.7326/0003-4819-149-10-200811180-00003

[bib79] Holland DP, Sanders GD, Hamilton CD et al. Costs and cost-effectiveness of four treatment regimens for latent tuberculosis infection. Am J Respir Crit Care Med 2009; 179: 1055–1060. 19299495 10.1164/rccm.200901-0153OC PMC2689913

[bib80] Joint Tuberculosis Committee of the British Thoracic Society. Control and prevention of tuberculosis in the United Kingdom: code of practice 2000. Thorax 2000; 55: 887–901. 11050256 10.1136/thorax.55.11.887 PMC1745632

[bib81] Sterling TR, Villarino ME, Borisov AS et al. Three months of rifapentine and isoniazid for latent tuberculosis infection. N Engl J Med 2011; 365: 2155–2166. 22150035 10.1056/NEJMoa1104875

[bib82] Sterling TR, Moro RN, Borisov AS et al. Flu-like and other systemic drug reactions among persons receiving weekly rifapentine plus isoniazid or daily isoniazid for treatment of latent tuberculosis infection in the PREVENT tuberculosis study. Clin Infect Dis 2015; 61: 527–535. 25904367 10.1093/cid/civ323 PMC4560029

[bib83] Gordin F, Chaisson RE, Matts JP et al. Rifampin and pyrazinamide vs isoniazid for prevention of tuberculosis in HIV-infected persons: an international randomized trial. Terry Beirn Community Programs for Clinical Research on Aids, the Adult Aids Clinical Trials Group, the Pan American Health Organization, and the Centers for Disease Control and Prevention Study Group. JAMA 2000; 283: 1445–1450. 10732934 10.1001/jama.283.11.1445

[bib84] Halsey NA, Coberly JS, Desormeaux J et al. Randomised trial of isoniazid versus rifampicin and pyrazinamide for prevention of tuberculosis in HIV-1 infection. Lancet 1998; 351: 786–792. 9519950 10.1016/S0140-6736(97)06532-X

[bib85] From the Centers for Disease Control and Prevention. Fatal and Severe Hepatitis Associated with Rifampin and Pyrazinamide for the Treatment of Latent Tuberculosis Infection–New York and Georgia, 2000. JAMA 2001; 285: 2572–2573. 11396466

[bib86] From the Centers for Disease Control and Prevention. Update: Fatal and Severe Liver Injuries Associated with Rifampin and Pyrazinamide for Latent Tuberculosis Infection, and Revisions in American Thoracic Society/CDC Recommendations–United States, 2001. JAMA 2001; 286: 1445–1446. 11596606

[bib87] Centers for Disease Control and Prevention, American Thoracic Society. Update: adverse event data and revised. American Thoracic Society/CDC recommendations against the use of rifampin and pyrazinamide for treatment of latent tuberculosis infection–United States, 2003. MMWR Morb Mortal Wkly Rep 2003; 52: 735–739. 12904741

[bib88] Pape JW, Jean SS, Ho JL et al. Effect of isoniazid prophylaxis on incidence of active tuberculosis and progression of HIV infection. Lancet 1993; 342: 268–272. 8101302 10.1016/0140-6736(93)91817-6

[bib89] Grant AD, Charalambous S, Fielding KL et al. Effect of routine isoniazid preventive therapy on tuberculosis incidence among HIV-infected men in South Africa: a novel randomized incremental recruitment study. JAMA 2005; 293: 2719–2725. 15941800 10.1001/jama.293.22.2719

[bib90] Whalen CC, Johnson JL, Okwera A et al. A trial of three regimens to prevent tuberculosis in ugandan adults infected with the human immunodeficiency virus. Uganda-Case Western Reserve University Research Collaboration. N Engl J Med 1997; 337: 801–808. 9295239 10.1056/NEJM199709183371201

[bib91] Rangaka MX, Wilkinson RJ, Boulle A et al. Isoniazid plus antiretroviral therapy to prevent tuberculosis: a randomised double-blind, placebo-controlled trial. Lancet 2014; 384: 682–690. 24835842 10.1016/S0140-6736(14)60162-8 PMC4233253

[bib92] Bucher HC, Griffith LE, Guyatt GH et al. Isoniazid prophylaxis for tuberculosis in HIV infection: a meta-analysis of randomized controlled trials. AIDS 1999; 13: 501–507. 10197379 10.1097/00002030-199903110-00009

[bib93] Samandari T, Agizew TB, Nyirenda S et al. 6-month versus 36-month isoniazid preventive treatment for tuberculosis in adults with HIV infection in Botswana: a randomised, double-blind, placebo-controlled trial. Lancet 2011; 377: 1588–1598. 21492926 10.1016/S0140-6736(11)60204-3

[bib94] Martinson NA, Barnes GL, Moulton LH et al. New regimens to prevent tuberculosis in adults with HIV infection. N Engl J Med 2011; 365: 11–20. 21732833 10.1056/NEJMoa1005136 PMC3407678

[bib95] John GT, Thomas PP, Thomas M et al. A double-blind randomized controlled trial of primary isoniazid prophylaxis in dialysis and transplant patients. Transplantation 1994; 57: 1683–1684. 8009608

[bib96] Naqvi R, Naqvi A, Akhtar S et al. Use of isoniazid chemoprophylaxis in renal transplant recipients. Nephrol Dial Transplant 2010; 25: 634–637. 19783599 10.1093/ndt/gfp489

[bib97] de Lemos AS, Vieira MA, Halpern M et al. Results of implementation of preventive recommendations for tuberculosis after renal transplantation in an endemic area. Am J Transplant 2013; 13: 3230–3235. 24119248 10.1111/ajt.12470

[bib98] Currie AC, Knight SR, Morris PJ. Tuberculosis in renal transplant recipients: the evidence for prophylaxis. Transplantation 2010; 90: 695–704. 20647975 10.1097/TP.0b013e3181ecea8d

[bib99] Sichletidis L, Settas L, Spyratos D et al. Tuberculosis in patients receiving anti-TNF agents despite chemoprophylaxis. Int J Tuberc Lung Dis 2006; 10: 1127–1132. 17044206

[bib100] Seong SS, Choi CB, Woo JH et al. Incidence of tuberculosis in Korean patients with rheumatoid arthritis (RA): effects of RA itself and of tumor necrosis factor blockers. J Rheumatol 2007; 34: 706–711. 17309133

[bib101] Gomez-Reino JJ, Carmona L, Angel Descalzo M et al. Risk of tuberculosis in patients treated with tumor necrosis factor antagonists due to incomplete prevention of reactivation of latent infection. Arthritis Rheum 2007; 57: 756–761. 17530674 10.1002/art.22768

[bib102] Tam LS, Leung CC, Ying SK et al. Risk of tuberculosis in patients with rheumatoid arthritis in Hong Kong–the role of TNF blockers in an area of high tuberculosis burden. Clin Exp Rheumatol 2010; 28: 679–685. 20822708

[bib103] van der Werf MJ, Langendam MW, Sandgren A et al. Lack of evidence to support policy development for management of contacts of multidrug-resistant tuberculosis patients: two systematic reviews. Int J Tuberc Lung Dis 2012; 16: 288–296. 22640442 10.5588/ijtld.11.0437

[bib104] Fraser A, Paul M, Attamna A et al. Drugs for preventing tuberculosis in people at risk of multiple-drug-resistant pulmonary tuberculosis. Cochrane Database Syst Rev 2006; 2: CD005435. 16625639 10.1002/14651858.CD005435.pub2 PMC6532726

[bib105] Bamrah S, Brostrom R, Dorina F et al. Treatment for LTBI in contacts of MDR-TB patients, Federated States of Micronesia, 2009–2012. Int J Tuberc Lung Dis 2014; 18: 912–918. 25199004 10.5588/ijtld.13.0028 PMC4730114

[bib106] Vikrant S, Agarwal SK, Gupta S et al. Prospective randomized control trial of isoniazid chemoprophylaxis during renal replacement therapy. Transpl Infect Dis 2005; 7: 99–108. 16390397 10.1111/j.1399-3062.2005.00103.x

